# Facile synthesis and characterization of magnetic nanocomposite ZnO/CoFe_2_O_4_ hetero-structure for rapid photocatalytic degradation of imidacloprid

**DOI:** 10.1016/j.heliyon.2019.e02870

**Published:** 2019-11-23

**Authors:** Matin Naghizadeh, Mohammad Ali Taher, Ali-Mohammad Tamaddon

**Affiliations:** a Department of Chemistry, Shahid Bahonar University of Kerman, Kerman, Iran; b Young Researchers Society, Shahid Bahonar University of Kerman, Kerman, Iran; c Center for Nanotechnology in Drug Delivery, Shiraz University of Medical Sciences, Shiraz, Iran; d Department of Pharmaceutics, School of Pharmacy, Shiraz University of Medical Sciences, Shiraz, Iran

**Keywords:** Analytical chemistry, Environmental science, Materials chemistry, ZnO/CoFe_2_O_4_ magnetic nanocomposite, Imidacloprid, Photocatalytic degradation, Water pollution

## Abstract

This work has attempted to investigate the potential of ZnO/CoFe_2_O_4_ magnetic nanocomposite to mineralize imidacloprid completely to have sustainable pollutant free and safe water supply. The co-precipitation method was performed to prepare the composites; was performed to characterize composites, scanning electron microscope (SEM), transmission electron microscopy (TEM), energy dispersive x-ray crystallography (EDX), x-ray diffraction (XRD), Fourier transform infrared (FT-IR) spectroscopy, and vibrating sample magnetometer (VSM). It was attempted to explore and enhance parameters influencing the process and the percentage of imidacloprid degradation, including photocatalyst amount, pesticide concentration, pH, radiation time, and temperature. UV-Vis spectrophotometer was used for the degradation percent of organochlorine pesticides. Parameters affecting the process, including photocatalyst amount, pesticide concentration, pH, radiation time, and temperature effect on the percentage of imidacloprid degradation were Investigated and optimized. 0.05 g of photocatalyst, with a concentration of 5 ppm for 45 min under light exposure was obtained at pH 10 at room temperature.

## 1 Introduction

A big problem in the world is the pollution of water, hence it is necessary to evaluate and review water resource policies. It is believed that food demands will be doubled globally within the next 50 years [1]. The food production has been boosted by applying in agriculture through managing many pests at various stages of crop production and storage; however, it polluted the soil and groundwater severely [2, 3, 4]. Some studies revealed that around 2.5 million tons of pesticides are used globally every year. In Iran, 60 thousand tons of pesticides are used annually, which is increasing at an annual rate of about 6–15%. Surprisingly, only 0.1 % of pesticides target the crops and, 99.9 % is wasted through the air, soil, and water, which leads to pollution inevitably [5]. Therefore, pesticides rank among the most common pollutants in surface and ground waters of agricultural areas. Human health is significantly influenced by the presence of pesticides in drinking water, and some of these pesticides are known to be carcinogens. They not only influence the people involved in agriculture but also are carried to long distances because of their longer half-lives and entering the water bodies in the end [6, 7, 8, 9, 10]. This scenario is worsened in countries like Iran due to limited water resources and existing resources being polluted by the reckless and unnecessary use of the agrochemicals [11]. Therefore, developing a well-organized and cost-effective technology seems essential to remove these dangerous pollutants from waters and wastewaters completely. Some methods have been proposed for removal of the pollutants from the waters and wastewaters such as adsorption, filtration, chemical oxidation, and biological treatments [12, 13]. Recently, the semiconductor photocatalysis has been favored because it considers the solution of environmental problems. These semiconductor photocatalysts generate electron and hole pairs (e^−^-h^+^) by irradiation of light energy that could be used to start oxidation and decrease reactions of the pesticide, respectively [14]. The number of photons striking the photocatalyst controls the reaction rate. One of the frequently studied photo-catalyst is ZnO due to its chemical stability, availability, and is low-cost. Besides, it is similar to TiO_2_ in terms of the energy band gap [15]. Cobalt, iron, and zinc metals were successfully used to decrease the half-lives of pesticides such as chlorpyrifos, imidacloprid, etc. These metals are capable of boosting the degradation rate and thus could be a catalyst for the degradation. Light energy can be collected by semiconductors like TiO_2_/ZnO for photo-degradation. Both of them are capable of boosting the degradation rate if combined in composite form through the reduction of the energy consumption for degradation and gathering additional energy. Suitable photocatalysts are selective, active, and highly stable; it is possible to re-recover and separate them effortlessly from the reaction medium. The industrial application of nanotechnology has expanded the production of nanocatalysts. The miniature size of the nanocomposites is not beneficial due to isolation issue; thus, when magnetic nanoparticles are used as nano photocatalyst, isolating and reusing the nanocomposites would be feasible at the end of the reaction using a suitable magnetic field. These magnetic nanophotocatalysts can substitute heterogeneous and homogenous catalysts because of separation capability and high levels and activity. In this paper, the co-precipitation method was applied to prepare ZnO/CoFe_2_O_4_ magnetic nanocomposite. This method has numerous advantages including: (1) the acidification treatment was used to increase the specific surface area of ZnO, and to ease post-functionalization, oxygen-containing functional groups were proposed as new adsorption and active sites; (2) the combination of CoFe_2_O_4_ separated photogenerated electrons and to photo-less, thus, the photocatalytic activity of the composite material was enhanced; and (3) CoFe_2_O_4_ supported on ZnO also provided new adsorption sites to boost the materials in terms of the catalytic performance. Besides, the activation of ZnO/CoFe_2_O_4_ magnetic nanocomposite under the irradiation containing visible light would result in its synergistic catalytic role with the CoFe_2_O_4_ leading to enhanced photocatalytic activity of the composite material. Imidacloprid as neonicotinoid insecticide has been used frequently in agricultural production; they are identified in the environment because they are soluble in water. Photolysis and hydrolysis are the main natural degradation modes of imidacloprid [16]. Therefore, this compound was selected to act the organic contaminants to exam the photocatalytic activity of the as-prepared ZnO/CoFe_2_O_4_ photocatalysts under visible light irradiation [17, 18].

## 2 Material and methods

### 2.1 Materials and apparatus

**Fig. 1:**
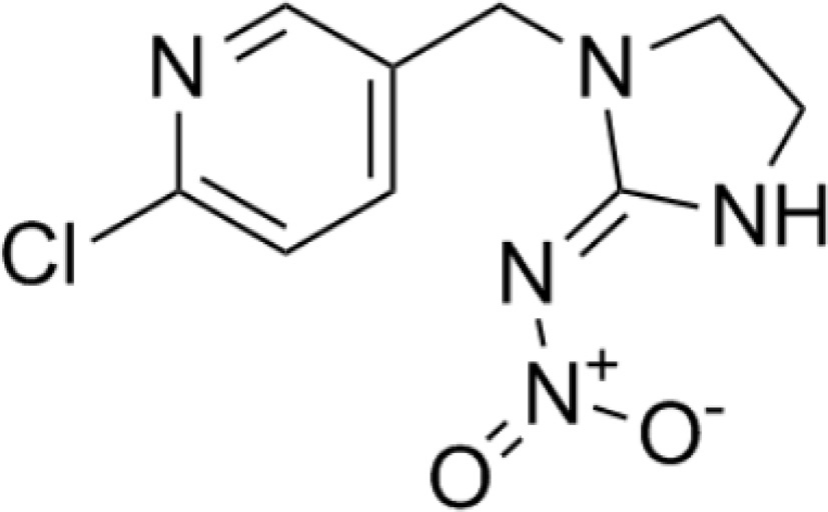
Imidacloprid structure.

In the present study, chemicals including zinc acetate, ammonium oxalate, absolute ethanol, ammonia solution, FeCl_3_.6H_2_O (98 %), and CoCl_2_.6H_2_O (98%) were analytical grades and purchased from Merck Company. Imidacloprid was purchased from Sigma. Fig. 1 shows the structure of the imidacloprid.

Distilled water was used for all solutions. An electronic balance Mettler AE-160 (Greifensee, www.mt.com) was used to weigh the samples. Varian Cary 50 spectrophotometer made in Australia. A Metrohm 827 pH-meter equipped with a consolidated glass–calomel electrode was used for pH calibration. To make the samples homogenized, magnetic stirrer hot plate (2000 rpm) and oven model 100 (Memmert, www.memmert.com) were used. A Sonorex digitec model DT 225H with 35 kHz ultrasonicator (Bandelin (www.bandelin.com), was applied for separating the nanoparticles in solutions. A Tensor 27 spectrometer (Bruker, www.bruker.com) was used to have the Fourier transform infrared (FT-IR) spectra. EM10C transmission electron microscope (TEM), (Zeiss, www.zeiss.com), was used with an accelerating voltage of 100 kV. Field emission-scanning electron microscopy (FE-SEM) images were obtained on a model Sigma (Zeiss, www.zeiss.com). The powder X-ray diffraction (XRD) patterns were examined on a model X'PertPro diffractometer (Panalytical, www.panalytical.com) using Cu Kα radiation in the 2ɵ range 10**–**80°. A vibrating sample magnetometer (VSM) model MDKFD (www.lakeshore.com) was used for magnetic measurements.

### 2.2 Preparation of photocatalyst

#### 2.2.1 Synthesis of ZnO nanoparticles

A new precipitation method was used to prepare ZnO NPs. 100 mL of distilled water was used to dissolve 0.01 M Zinc acetate (2.19 g). After precise weighing, 0.01 M of solid ammonium oxalate (1.42 g) powder was added to the zinc acetate solution. To dissolve the ammonium oxalate completely, the suspension was stirred. To increase the pH of the solution to 10, the ammonia solution was added in drops. The formation of a white precipitate of zinc oxalate was observed afterward. The magnetite precipitates were collected by a magnet after washing several times with deionized water and then with ethanol. The wet zinc oxalate particles obtained were allowed to dry in air for 24 h; then it was dried in an oven at 110 °C for 90 min. The dry powder obtained was carefully gathered in silica crucible and heated in a muffle furnace at 700 °C for about 3 h. During calcination, zinc oxalate was decomposed according to the following equation leading to the formation of ZnO NPs [19].

$$\underset{\text{Zinc acetate}}{\text{Zn}{\left({\text{CH}}_{3}\text{COO}\right)}_{2}}+\underset{\text{ammonium oxalate}}{{\left({\text{COONH}}_{4}\right)}_{2}}\overset{\text{pH }=\;10\text{ / }{\text{NH}}_{4}\text{OH}}{\to }{\text{ZnC}}_{2}{\text{O}}_{2}{+\text{CH}}_{3}{\text{COOH}+\text{H}}_{2}{\text{O}+\text{N}}_{2}↑$$

$${2\text{ZnC}}_{2}{\text{O}}_{4}{+\text{O}}_{2}\overset{700{}^{\circ}\text{C}}{\to }{2\text{ZnO}+4\text{CO}}_{2}↑$$

#### 2.2.2 Preparation of ZnO/CoFe_2_O_4_ magnetic nanocomposite

FeCl_3_.6H_2_O (1.08 g) and CoCl_2_·6H_2_O (0.48 g) were dissolved in 100 mL deionized water to prepare CoFe_2_O_4_ magnetic nanoparticles. Then, stirring was applied to this solution using a mechanical stirrer (1000 rpm) at 80 °C for 1 h. Oxygen was expelled through continuous bubbling of Nitrogen in this solution to expel oxygen. Afterward, 12 mL of 25% NH_3_ was quickly added to the solution. Then, the bulk solution turned from orange to brown straight away. After washing several times first with deionized water and second with ethanol, a magnet was used to collect the magnetite precipitates. Ultimately, they were put to dry in an oven at 80 °C for 6 h followed by calcination at 350 °C for 3 h to form nanocrystalline ZnO/CoFe_2_O_4_.

### 2.3 Photocatalytic degradation

To explore the photocatalytic degradation of the synthesized nanocomposite, the experiments were performed as follows: 0.05 g of the weighed composition was poured into 5 mL of a 10-ppm solution of imidacloprid pesticide, and the solution was put under direct exposure to visible light and was stirred for 30 min. Then, UV-Vis spectroscopy was applied to explore the reaction mixture in terms of studying the degradation of imidacloprid and determining the amount of imidacloprid pesticide remaining in the solution. This process was done for all stages of final catalyst synthesis, and Table 1 shows its degradation percentage.

**Table 1:** Percent degradation of imidacloprid in all stages of final catalyst synthesis.

Catalyst	Degradation percentage
ZnO nanoparticles	56.24 ± 2
CoFe_2_O_4_ magnetic nanoparticles	58.45 ± 2
ZnO/CoFe_2_O_4_ magnetic nanocomposite	98.11 ± 1

**Scheme 1:**
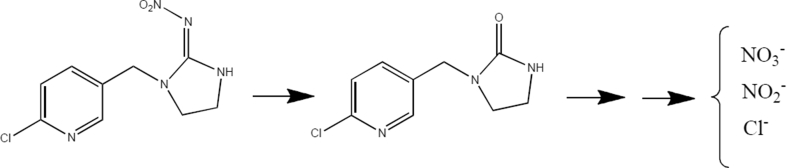
Proposed mechanism of imidacloprid degradation.

Scheme 1 depicts the proposed mechanism of imidacloprid degradation [20, 21, 22]. As shown, the products of degradation have been turned into inorganic compounds; besides, UV peak was not observed in a region of 254 nm; thus, no organic intermediates were logged as reported in the literature [23]. The products of decomposition were Cl^−^, NO_3_^-,^ and NO_2_^-^. The tests for halide ions using silver nitrate solution confirmed the presence of Cl^−^ and the method in our research group [24] was used to verify the presence of nitrate and nitrite.

## 3 Results and discussion

### 3.1 Characteristics of synthesized nanoparticles

#### 3.1.1 Fourier transforms infrared spectroscopy (FT-IR) analysis

**Fig. 2:**
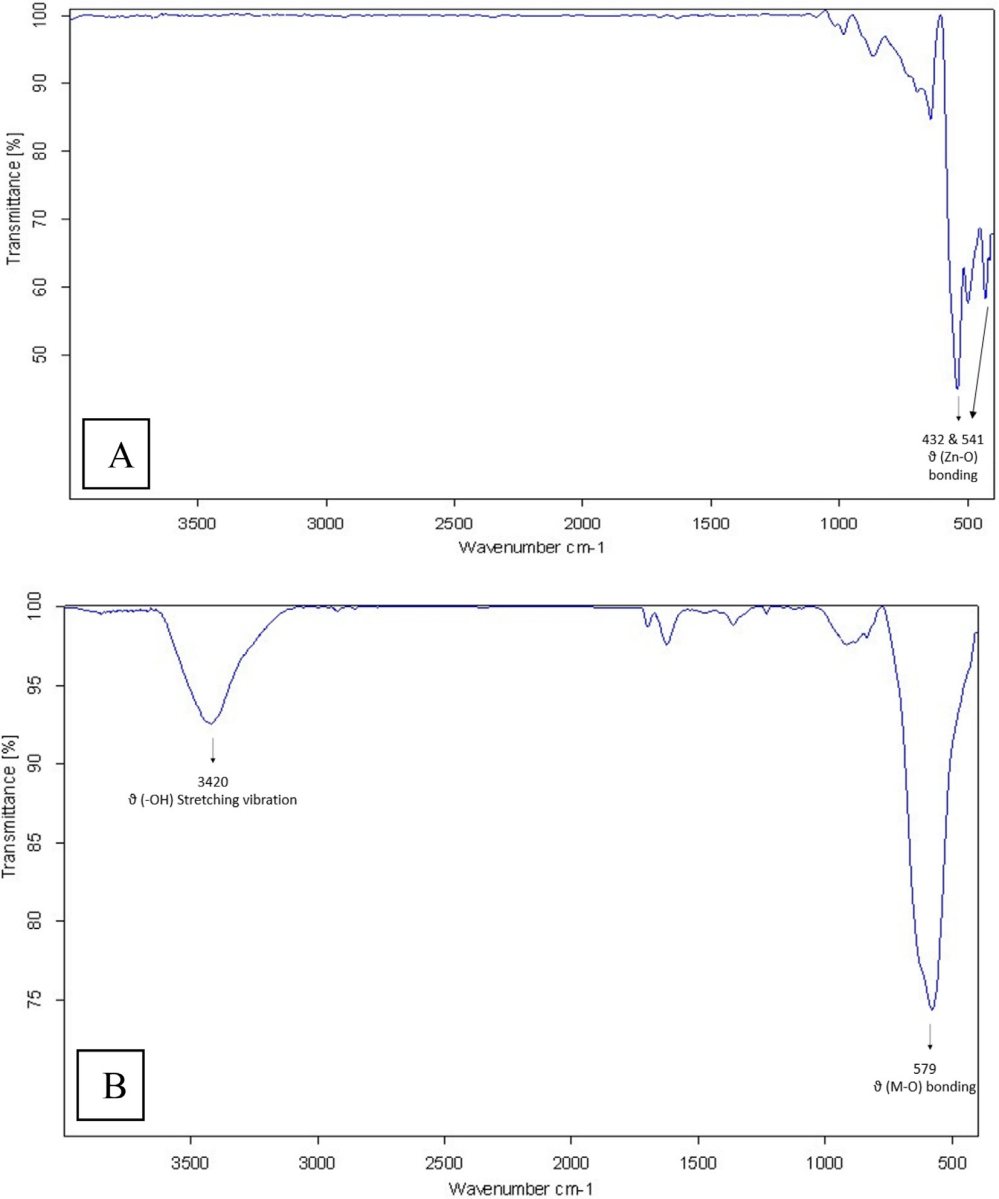
FT-IR spectra of (A) ZnO and (B) ZnO/CoFe_2_O_4_ nanoparticles.

As shown in Fig. 2A FTIR spectrum provides data on the chemical bonding between Zn and O. The spectrum indicated a broad peak of 432 cm^−1^ and shoulder 541 cm^−1^ corresponding to ZnO nanoparticle. The remaining spectrum was smooth with a few peaks of CO_2_. Fig. 2B shows IR spectra of the co-precipitated ZnO/CoFe_2_O_4_ powders. The absorption band at 579 cm^−1^ is associated with the stretching vibrations of the M-O bonds in the tetrahedral site of spinel ferrite. The broadband between 3600 and 3300 cm^−1^ centered at 3420 cm^−1^ is the result of the O–H stretching vibration of physically adsorbed water onto the nanoparticle surface [25, 26].

#### 3.1.2 FESEM images

**Fig. 3:**
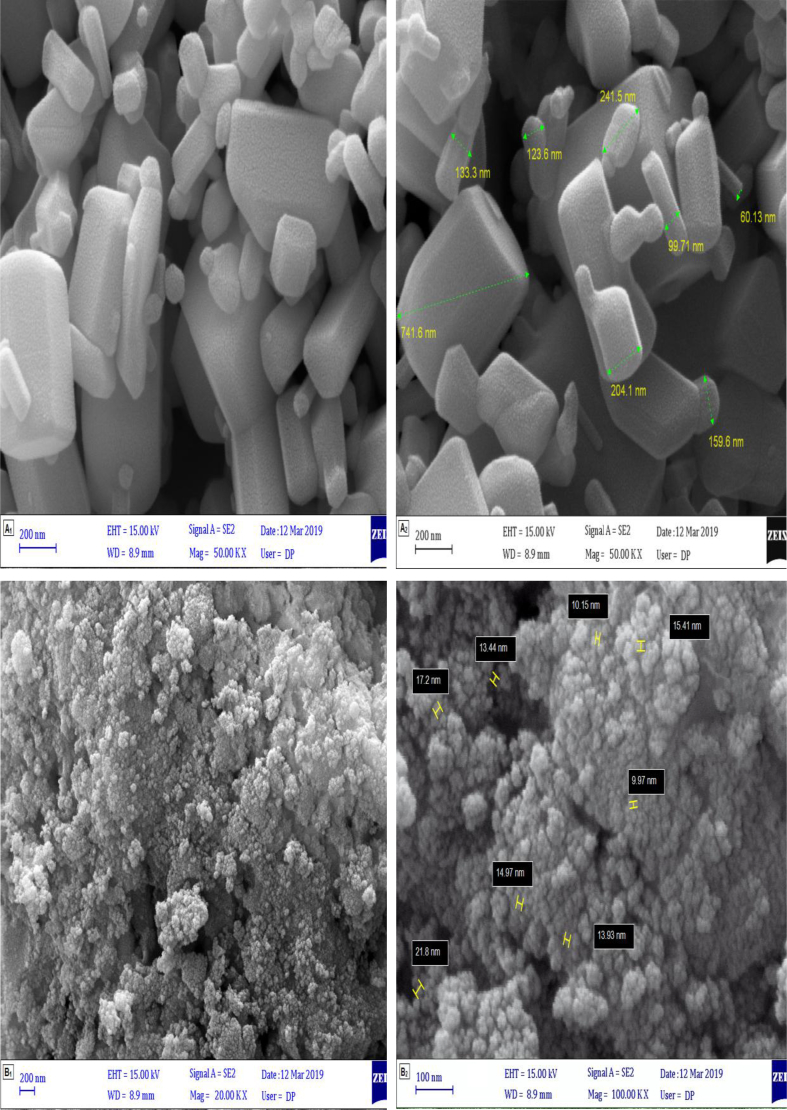
FESEM micrographs of prepared (A_1_ & A_2_) pure ZnO nanoparticles and (B_1_ & B_2_) ZnO/CoFe_2_O_4_ magnetic nanocomposite.

FE-SEM images were used to characterize the surface morphological images of the synthesized ZnO nanoparticles and ZnO/CoFe_2_O_4_ magnetic nanocomposite that were shown in different magnification. As shown in Fig. 3, the uniform morphology and hexagonal rods shape of the ZnO nanoparticles and spherical shape of CoFe_2_O_4_ nanoparticles dispersed on the ZnO nanoparticles are confirmed.

#### 3.1.3 EDX patterns

**Fig. 4:**
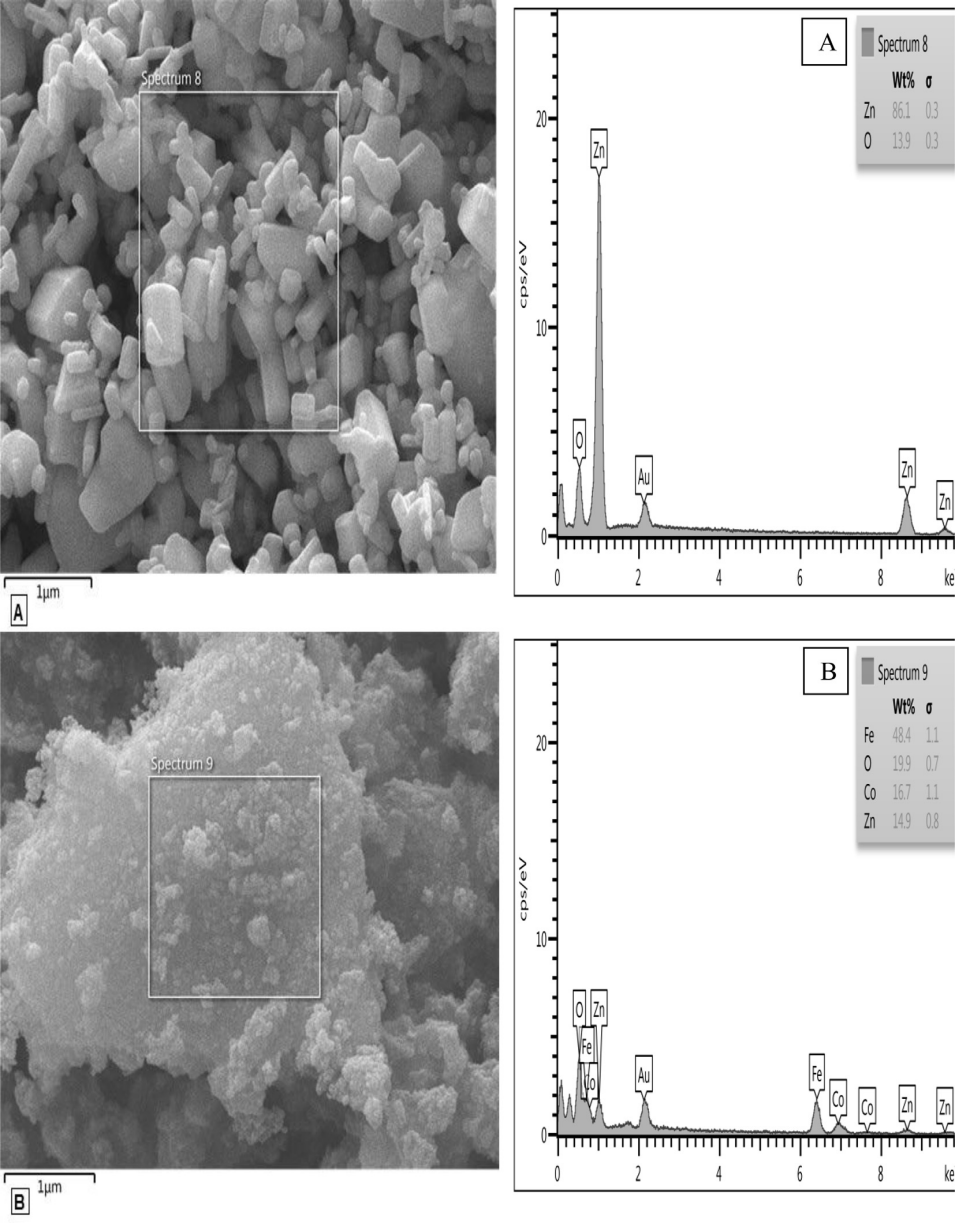
EDX patterns of prepared (A) pure ZnO nanoparticles and (B) ZnO/CoFe_2_O_4_ magnetic nanocomposite.

Fig. 4 shows the analysis of EDX to explain the elemental composition of the pure ZnO nanoparticles and ZnO/CoFe_2_O_4_ magnetic nanocomposite. As expected, zinc and oxygen are observed in the spectrum in Fig. 4A; iron, oxygen, cobalt, and zinc elements are observed in spectrum in Fig. 4B.

#### 3.1.4 TEM images

**Fig. 5:**
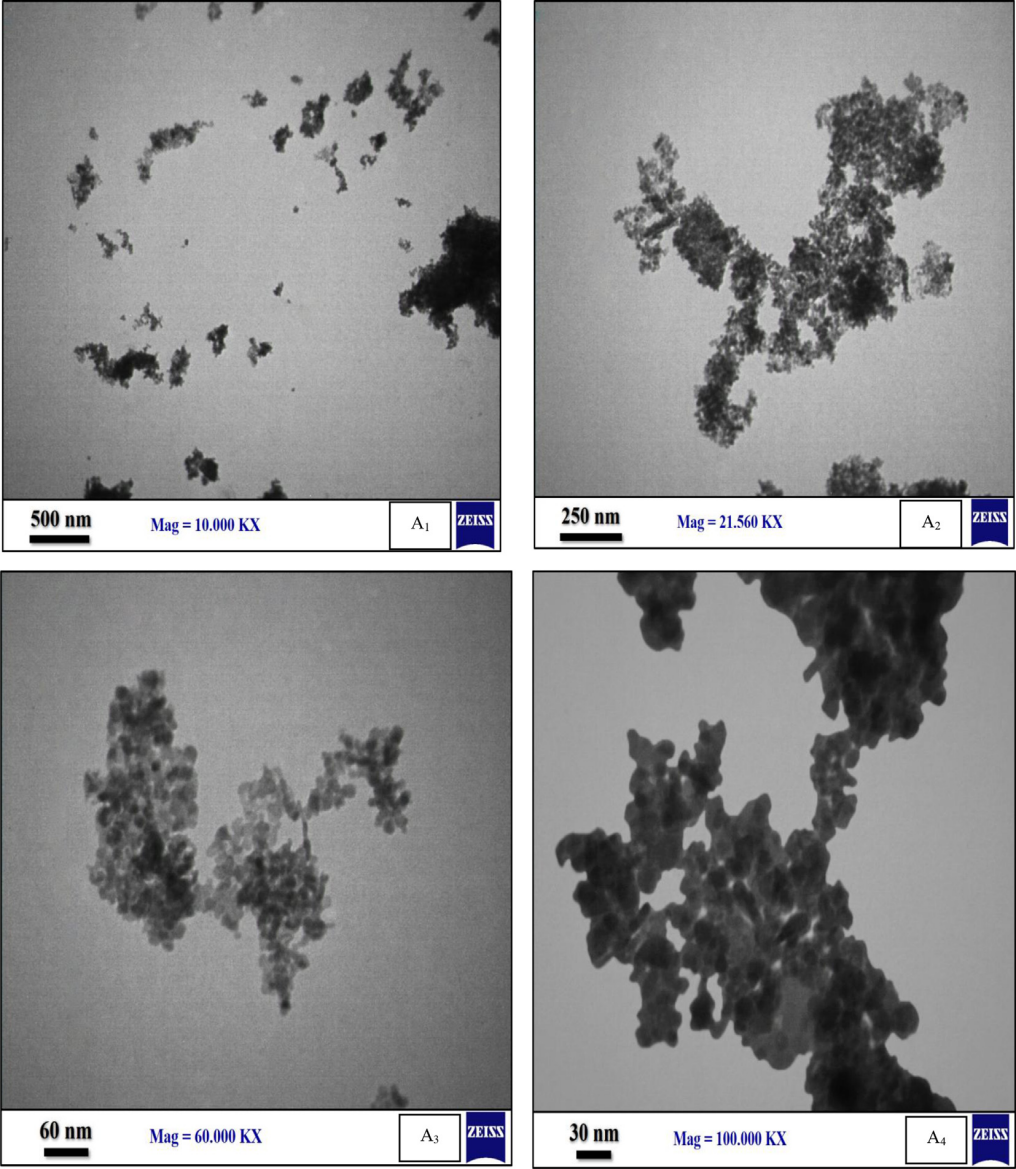
TEM micrograph (A_1_ - A_4_) of ZnO/CoFe_2_O_4_ magnetic nanocomposite.

As shown in Fig. 5, the fine dispersion of black particles (CoFe_2_O_4_) on the grey surface (ZnO) is verified by the TEM analysis of ZnO/CoFe_2_O_4_ magnetic nanocomposite. It is also assumed that the grain boundaries disperse the Co–Fe phase on the surface of ZnO. TEM Figure that the black dispersion confirmed the successful occurrences of CoFe_2_O_4_ in the ZnO/CoFe_2_O_4_ magnetic nanocomposite. Also, the diameter of the magnetic core is less than 10 nm.

#### 3.1.5 XRD patterns

**Fig. 6:**
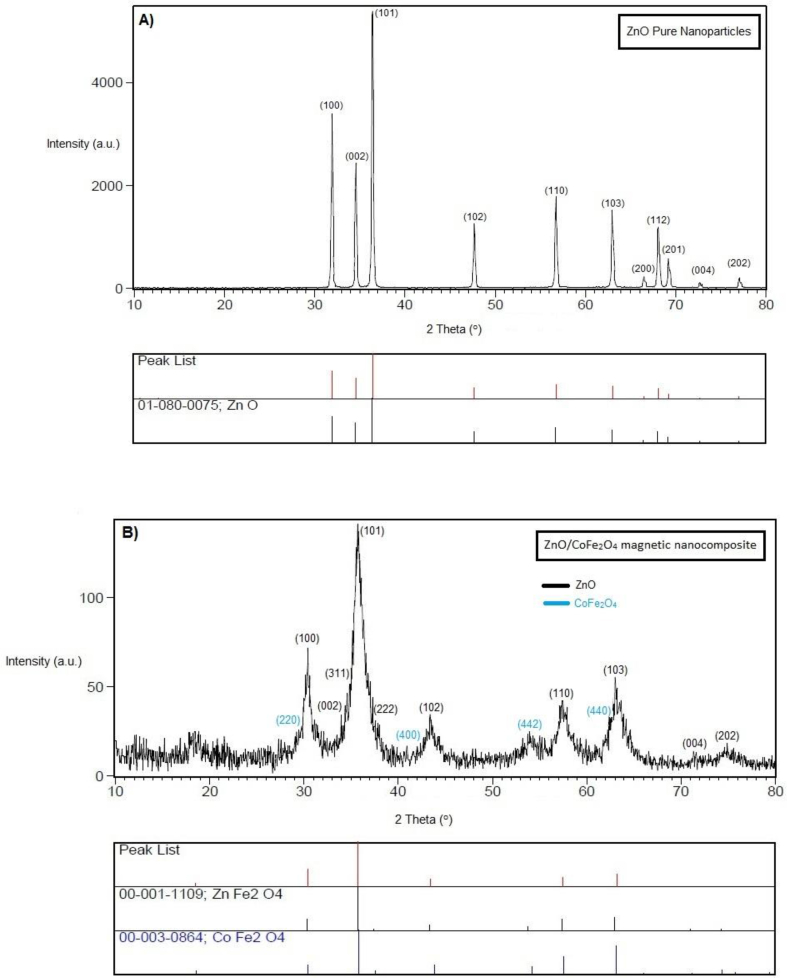
(A) XRD patterns from pure ZnO nanoparticles and (B) ZnO/CoFe_2_O_4_ magnetic nanocomposite.

Fig. 6 shows a usual XRD pattern of the ZnO nanoparticles and ZnO/CoFe_2_O_4_ magnetic nanocomposite. The diffraction peaks (2ϴ = 31.9°, 34.6°, 36.6°, 47.7°, 56.7°, 62.9°, 66.5°, 68.1°, 69.2°, 72.7°, 77.1°) related to (100), (002), (101), (102), (110) (103) (200) (112) (201) (004) (202), can be indexed to hexagonal structure of the ZnO nanoparticles with space group hexagonal P_63_mc. The diffraction peaks (2ϴ = 35. 7°, 43.4°, 57.5°, 63.2°) related to (220), (400), (442), (400) can be indexed to the cubic structure of the CoFe_2_O_4_ nanoparticles with space group cubic Fd_3_m. The highest peak (35. 7°) was chosen for the size calculation. According to the Debye-Scherrer equation crystal size of the ZnO/CoFe_2_O_4_ magnetic nanocomposite was 29 nm.

#### 3.1.6 Magnetic measurements

**Fig. 7:**
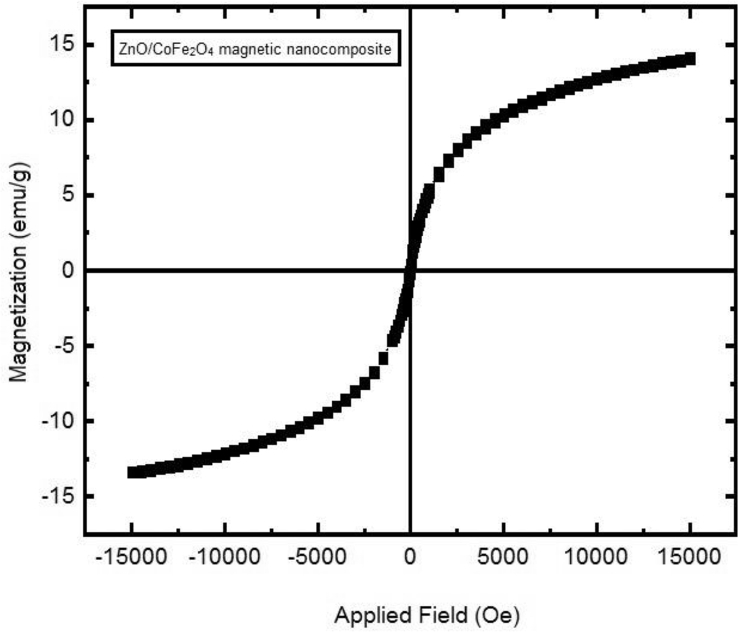
VSM curve of ZnO/CoFe_2_O_4_ magnetic nanocomposite.

As shown in Fig. 7, the saturation magnetization value was 15 emu/g. Moreover, a strong magnetic effect is made by ZnO/CoFe_2_O_4_ magnetic nanocomposite, and this characteristic could be applied for photocatalyst separation.

#### 3.1.7 UV-visible absorption spectra of ZnO and ZnO/CoFe_2_O_4_ nanoparticles

**Fig. 8:**
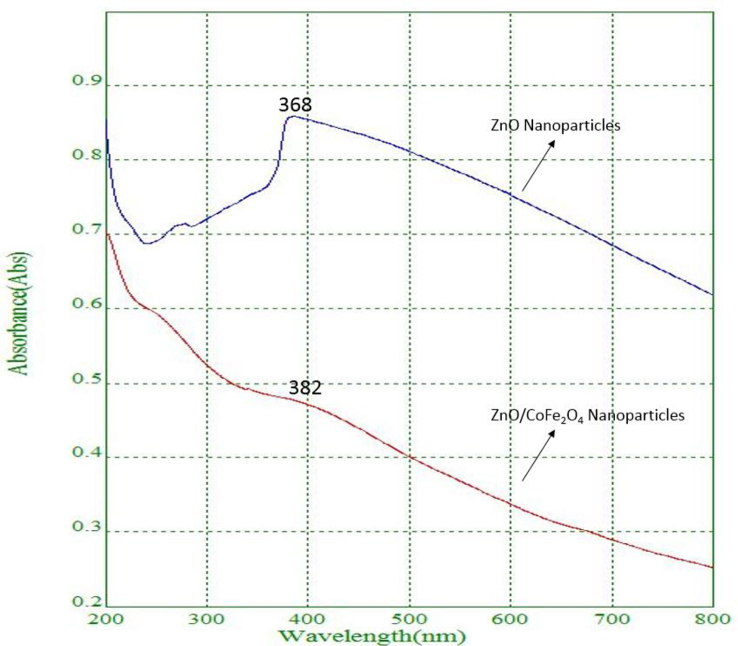
UV–Vis spectra for ZnO and ZnO/CoFe_2_O_4_ nanoparticles.

Fig. 8 shows the UV–visible absorption spectra of ZnO nanoparticles. The light source restricted our spectrometer, but a blue shift is observed in the absorption band of the ZnO nanoparticles because of the quantum confinement of the excitations present in the sample as compared with the bulk ZnO particles. This optical phenomenon implies that these nanoparticles have a quantum size effect. Fig. 8 shows UV–visible spectra absorbance peaks at 368 and 382 nm for the ZnO and CoFe_2_O_4_ nanoparticles dispersed in water [27].

### 3.2 Effect of different parameters on the degradation of imidacloprid pesticide

In this section, it was attempted to explore and enhance parameters influencing the process and the percentage of imidacloprid degradation, including photocatalyst amount, pesticide concentration, pH, radiation time, and temperature.

### 3.3 Photocatalytic degradation

#### 3.3.1 Effect of photocatalyst amount

**Fig. 9:**
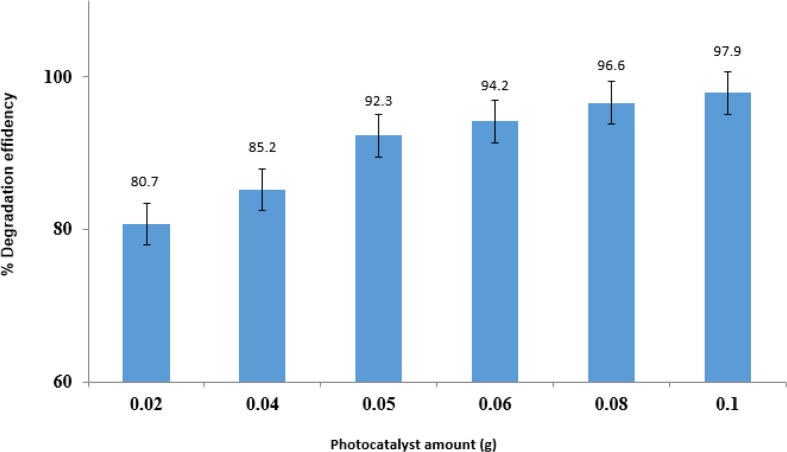
Effect of ZnO/CoFe_2_O_4_ nanoparticles dose in 10 mL imidacloprid pesticide.

It was also tried to show how the synthesized photocatalyst influences the degradation percentage of imidacloprid pesticide from 0.02 to 0.1 g. As shown in Fig. 9, an increased amount of ZnO/CoFe_2_O_4_ magnetic nanocomposite, leads to an increased percentage of imidacloprid pesticide degradation. Some adsorbed molecules cause the increased destruction rate on the photocatalyst surface; besides, more photons were observed [28, 29, 30]. Above the specified amount, the solution is opaque, the less light enters the solution, and the photocatalytic process is reduced [31].

#### 3.3.2 Imidacloprid concentration effect

**Fig. 10:**
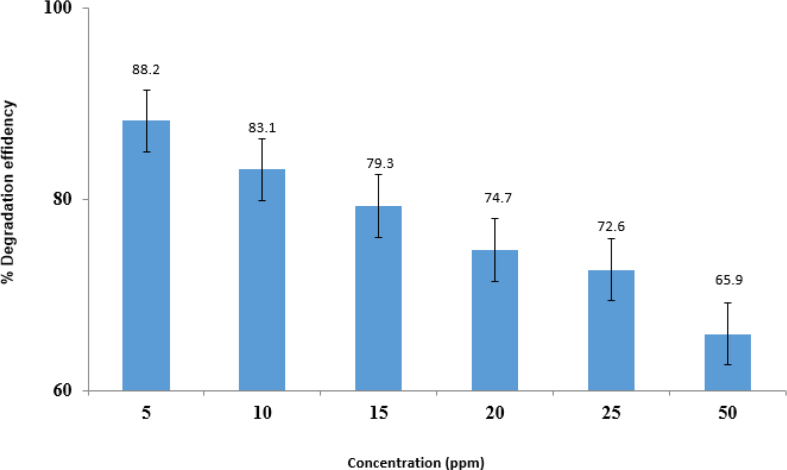
Effect of imidacloprid initial concentration.

Fig. 10 shows how the initial concentration of imidacloprid influences the degradation rate by changing the concentration from 5-50 ppm, keeping the time at 60 min with a photocatalyst amount of 0.05 g, and the degradation percentage. The increased concentration of imidacloprid pesticides decreased the degradation percentage. The formation of Hydroxyl radicals is the result of holes reacting with OH^−^ and H_2_O (through Radical trapping experiments). Separately, isopropyl alcohol, benzoquinone, silver nitrate, and potassium iodide were added to the pesticide solution in the presence of a catalyst. The results showed that after the addition of potassium iodide, the degradation process is decreased showing that the holes (h^+^) led to the water oxidation or the capture of an electron from the hydroxyl ion, which resulted in the pesticide degradation. Organochlorine ions replaced OH^−^ ions adsorbed on the photocatalyst surface. OH, radicals influence the rate of imidacloprid degradation. Hydroxyl radicals are decreased in concentrations higher than the optimal concentration of organochlorine compounds due to reduced active sites accessible for the production of OH radicals. Moreover, the increased concentration of the solution had a preventive role in light penetration through the screening effect [32, 33].

#### 3.3.3 Illumination time effect

**Fig. 11:**
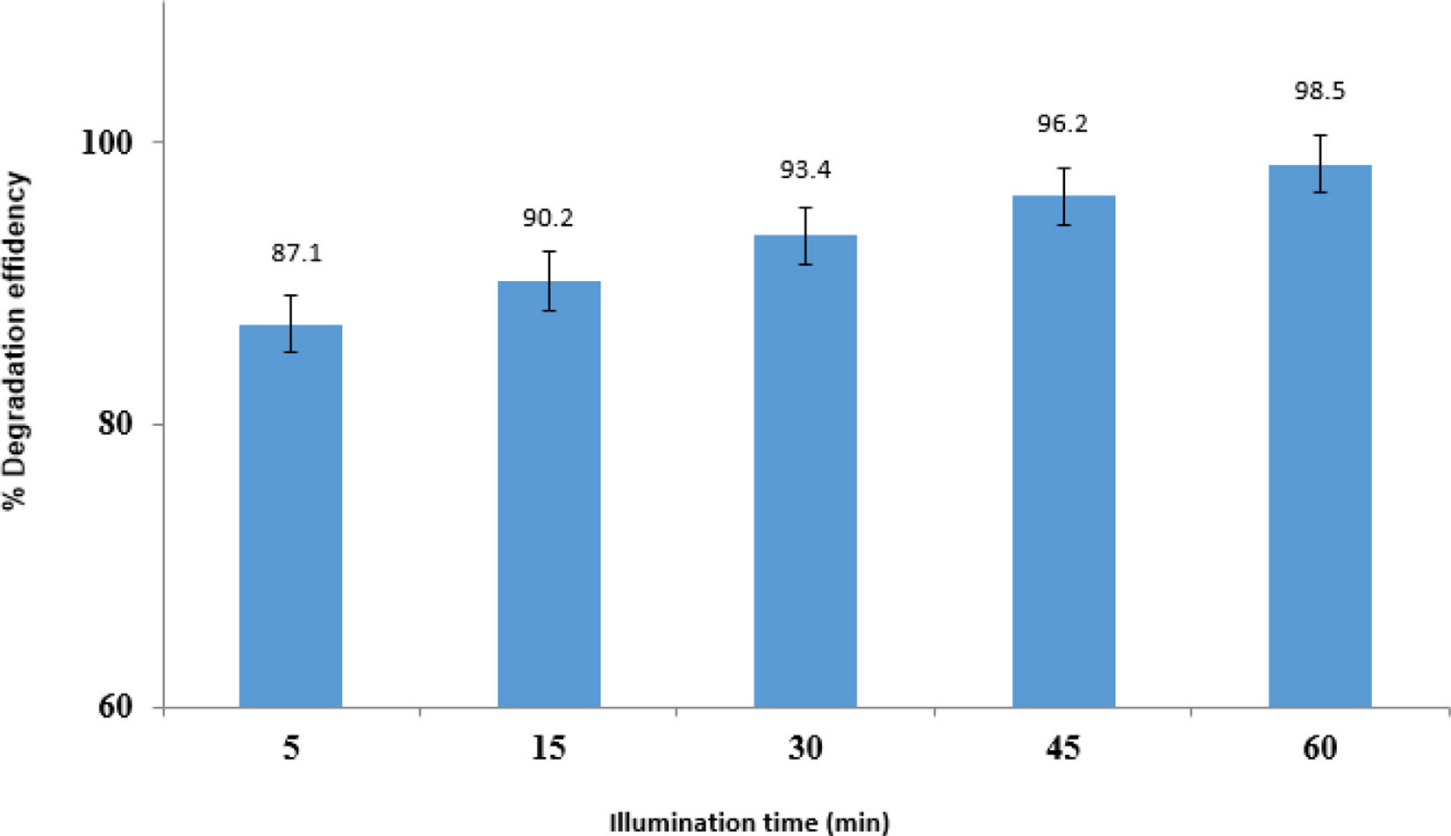
Effect of Illumination time.

Fig. 11 shows how irradiation time affects the percentage of degradation of imidacloprid in the range of 5–60 min in optimal conditions and the percentage degradation. As shown, increased radiation time with visible light (LED lamp 50 W) led to increased efficiency of photocatalytic degradation. The photocatalytic degradation of the imidacloprid pesticides reacts on the ZnO/CoFe_2_O_4_ magnetic nanocomposite surface. Since the photocatalytic process necessitates the presence of O_2_ and H_2_O, light irradiation on the ZnO/CoFe_2_O_4_ magnetic nanocomposite surface forms the electron-hole pairs. After the start of the reaction, they bind to the nanostructured sites very fast because of the high amount of imidacloprid; thus, the degradation is increased. 45 min was the optimal interval, according to the obtained data. The photocatalytic reactions are greatly influenced by pH.

#### 3.3.4 Effect of pH

**Fig. 12:**
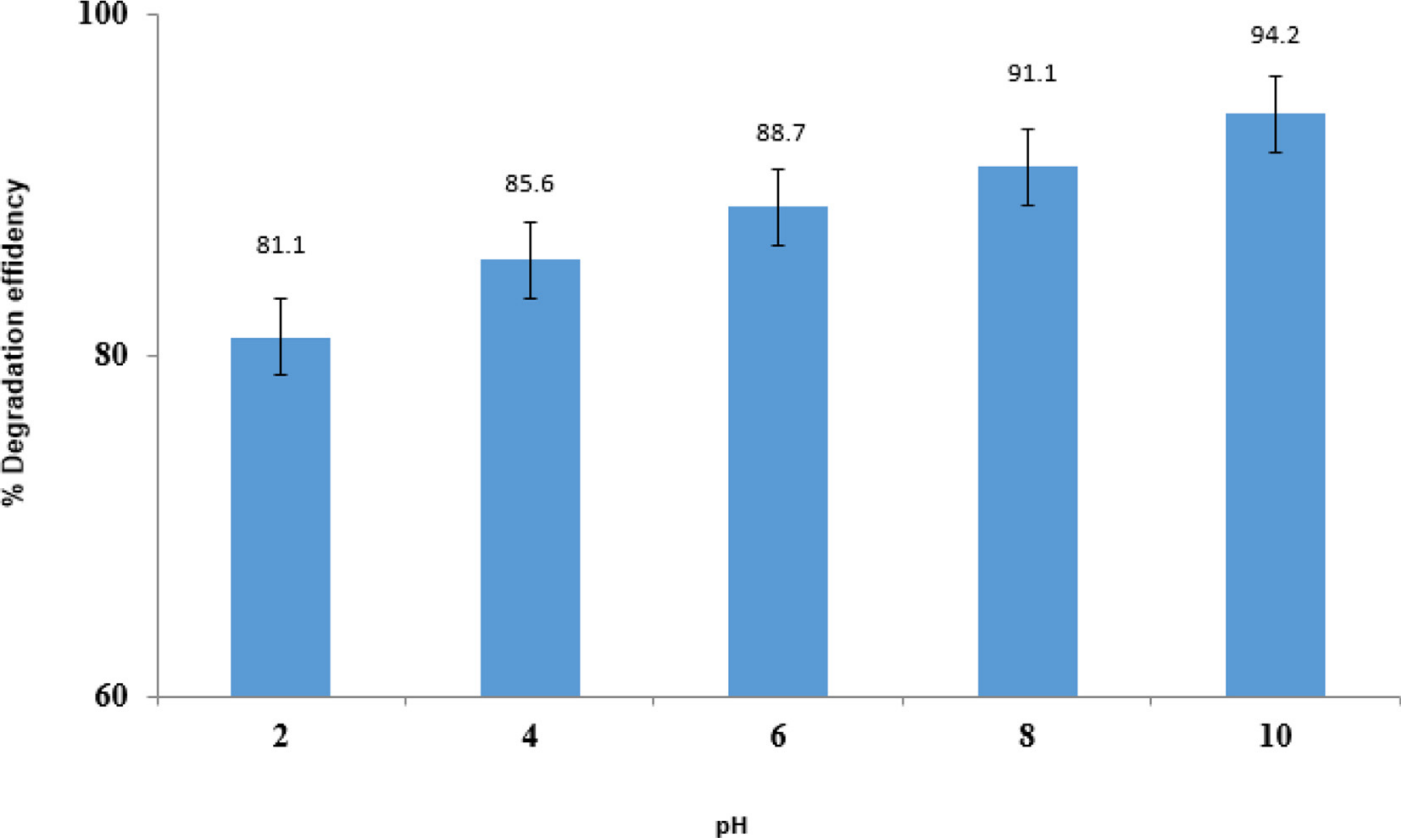
Effect of pH.

Fig. 12 shows the solutions reached under the optimal conditions with the pH range 2–10 and the photocatalytic degradation in the present study. As shown, at pH = 10, the best performance was observed due to the positive reaction of the hydroxide radicals formed on the catalyst surface positively with holes, and positive holes act as dominant species and oxidation sites at low pH; whereas hydroxide radicals in pH higher than the environment (pH > 7). Consequently, in the pH > 7, the formation of the hydroxide radicals took place on the surface of ZnO/CoFe_2_O_4_ magnetic nanocomposite leading to the degradation of imidacloprid pesticides.

#### 3.3.5 Effect of temperature

**Fig. 13:**
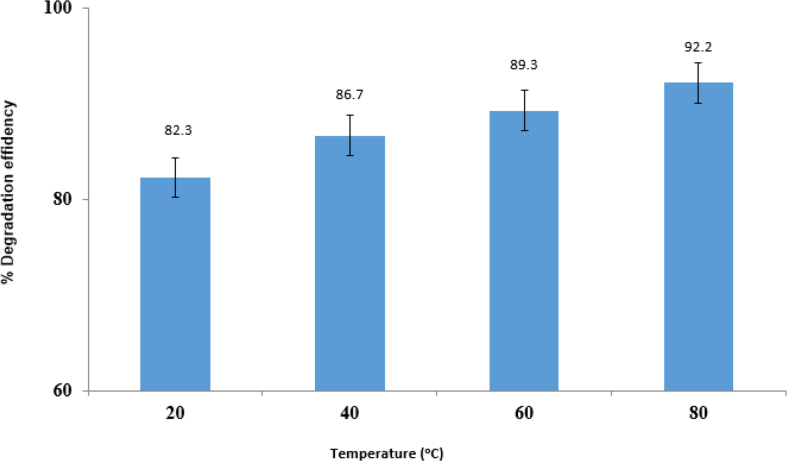
Effect of temperature.

The relationship between the photocatalytic efficacy of imidacloprid and temperature under.

optimal conditions was explored. As shown in Fig. 13, increasing temperature from 20-80 °C led to increased photocatalytic efficacy of imidacloprid. Besides, increasing the temperature, increased the percentage of degradation and the highest degradation rate was logged at 80 °C.

#### 3.3.6 Reuse of synthesized photocatalysts

**Fig. 14:**
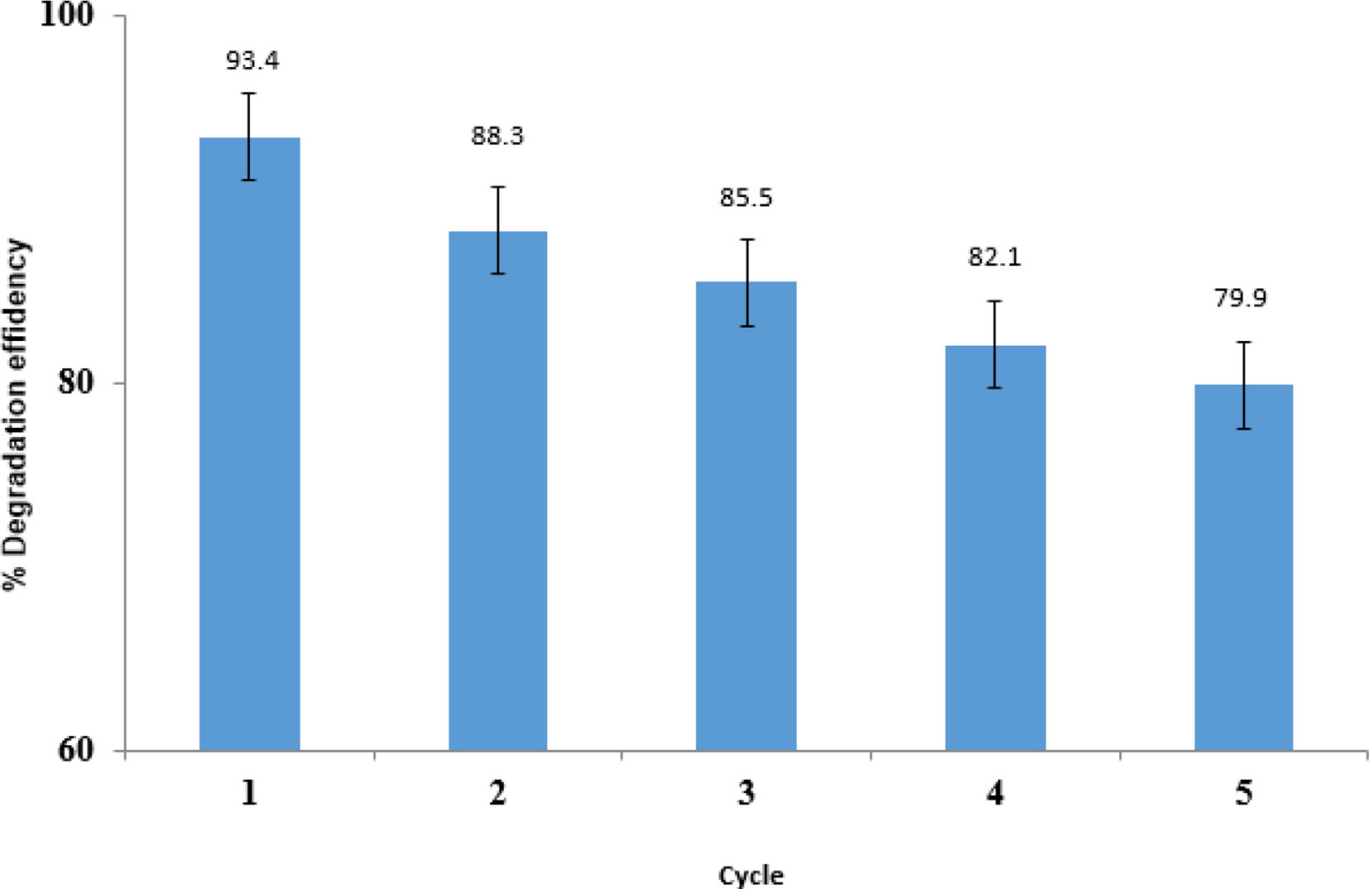
Effect of reuse of synthesized photocatalysts on the degradation of imidacloprid.

As a photocatalyst in the degradation of imidacloprid pesticide, ZnO/CoFe_2_O_4_ magnetic nanocomposite was examined in terms of sustainability and reuse by applying the optimal experimental conditions. The magnet was isolated from the solution after the photocatalyst test ended; then, the solution was washed thoroughly with distilled water and allowed to dry. According to Fig. 14, the percentage of degradation of the imidacloprid with the photocatalyst synthesized after about five times remains about to 80%. In other words, that is, it is possible to use the magnetic nanoscale photocatalyst again for at least five times without losing its effectiveness.

### 3.4 Comparison with other methods

Our nanocatalyst was compared to other photocatalysts for the degradation of imidacloprid pesticide and results are shown in Table 2.

**Table 2:** The photocatalytic degradation efficiency of imidacloprid with various photocatalysts.

Pesticide	Photocatalyst	Degradation percentage	References
Imidacloprid	Membrane	80 ± 2	[34]
Imidacloprid	GO@TiO2	93 ± 1	[35]
Imidacloprid	Photon-Fenton	95 ± 1	[36]
Imidacloprid	H3PW12O40/La–TiO2	98 ± 1	[37]
Imidacloprid	ZnO/CoFe_2_O_4_	98.1 ± 1	This work

## 4 Conclusion

Many photocatalysts have been used for the degradation of pesticides. Among them, a composite nanostructure was synthesized possessing photocatalytic properties, separation capability, high absorption capacity, etc., which could be used to mitigate environmental issues. FT-IR, FESEM, EDX, TEM, XRD, VSM, and UV-Visible were used to characterize the photocatalyst. According to the results of this work, the ZnO/CoFe_2_O_4_ magnetic nanocomposite could be an effective nano photocatalyst in the degradation of pesticides, including imidacloprid. For the degradation of pesticides, the photocatalytic activity of the synthesized nanostructure was boosted, and the results revealed that the optimum photocatalyst value could degrade 0.05 g of at least 5 ppm of pesticide at pH = 10. These results suggest that the ZnO/CoFe_2_O_4_ magnetic nanocomposite is very suitable for potential applications in degradation of pesticides.

## Declarations

### Author contribution statement

Matin Naghizadeh: Performed the experiments; Analyzed and interpreted the data; Wrote the paper.

Mohammad Ali Taher: Analyzed and interpreted the data; Wrote the paper.

Ali-Mohammad Tamaddon: Analyzed and interpreted the data.

### Funding statement

This research did not receive any specific grant from funding agencies in the public, commercial, or not-for-profit sectors.

### Competing interest statement

The authors declare no conflict of interest.

### Additional information

No additional information is available for this paper.
